# **Precancer: The Beginning and the End of Cancer.** Edited by Jules J. Berman, MD, PhD (E-Mail: jjberman1@comcast.net) with G. William Moore, MD, PhD. Jones & Bartlett Publishers: Sudbury, MA, USA, 2009; 186 pp; 42.95$; ISBN: 9780763777845

**DOI:** 10.3390/cancers1010021

**Published:** 2009-12-11

**Authors:** Kara L. Kuntz-Melcavage

**Affiliations:** National Institute of Neurological Disease and Stroke, National Institute of Stroke Diagnostics and Therapeutics Section, 10 Center Drive, Room B1D-733, MSC 1063, Bethesda, MD 20892-1063, USA; E-Mail: kuntzmelcavagkl@ninds.nih.gov

This concisely written book makes a powerful argument that the focus of research in the cancer field should be shifted from full-stage cancer to the more promising area of precancer. The entire book encompasses less than 200 pages, including an extensive glossary aimed at clarifying terminology with which a person lacking a research background may be unfamiliar. Because explanations of scientific terminology are located in the glossary, Berman and Moore have prevented the book from being too elementary for an advanced scientist. The logical organization of the chapters results in this becoming quite an easy book to read.

The authors present a powerful argument for their belief that cancer research should be refocused by first explaining the faults of current investigative and clinical approaches to cancer, progressing through a biological explanation of precancer, and concluding with thoughts about how more aggressive treatments for precancer could be a successful approach for eradicating cancer. Berman’s extensive experience as a pathologist, combined with his years of employment at the National Cancer Institute leaves little doubt that he is well qualified to write this book. Moore’s comparable background in pathology provides added credibility to the ideas presented in *Precancer*.

This book is especially relevant in light of recent debates regarding the importance of PAP smears and mammograms to young women. As described in *Precancer*, PAP smears are a technique for detecting precancer that is currently practiced and has proven to be successful for preventing cervical cancer. Considering the statistics presented regarding death rates from cervical cancer in countries in which PAP smears are routinely available compared with countries in which they are not widely used may impact one’s view towards increasing the age at which routine screens occur.

Berman and Moore’s book presents a persuasive argument for directing more attention towards precancer research. At times, it seems as though the preponderance of statistics and references supporting their idea may be presented in a biased view to support the authors’ thesis, but in a book designed to foster a new approach to cancer research an influential argument is expected. As a scientist who is unfamiliar with cancer, I found the book to be enjoyably educational and feel that the material is presented at a level that is appropriate for a general audience. Researchers who are more familiar with cancer research will likely derive pleasure from considering the author’s ideas and perhaps devising counterarguments. Given the speed with which this book can be read, I think it is well worth the time invested in reading it to gain familiarity with the concept of precancer and become appreciative of this powerful target for preventing cancer.

